# *Clostridium perfringens* α-toxin impairs erythropoiesis by inhibition of erythroid differentiation

**DOI:** 10.1038/s41598-017-05567-8

**Published:** 2017-07-12

**Authors:** Teruhisa Takagishi, Masaya Takehara, Soshi Seike, Kazuaki Miyamoto, Keiko Kobayashi, Masahiro Nagahama

**Affiliations:** Department of Microbiology, Faculty of Pharmaceutical Sciences, Tokushima Bunri University, Yamashiro-cho, Tokushima, 770-8514 Japan

## Abstract

*Clostridium perfringens* α-toxin induces hemolysis of erythrocytes from various species, but it has not been elucidated whether the toxin affects erythropoiesis. In this study, we treated bone marrow cells (BMCs) from mice with purified α-toxin and found that TER119^+^ erythroblasts were greatly decreased by the treatment. A variant α-toxin defective in enzymatic activities, phospholipase C and sphingomyelinase, had no effect on the population of erythroblasts, demonstrating that the decrease in erythroblasts was dependent of its enzymatic activities. α-Toxin reduced the CD71^+^TER119^+^ and CD71^–^TER119^+^ cell populations but not the CD71^+^TER119^−^ cell population. In addition, α-toxin decreased the number of colony-forming unit erythroid colonies but not burst-forming unit erythroid colonies, indicating that α-toxin preferentially reduced mature erythroid cells compared with immature cells. α-Toxin slightly increased annexinV^+^ cells in TER119^+^ cells. Additionally, simultaneous treatment of BMCs with α-toxin and erythropoietin greatly attenuated the reduction of TER119^+^ erythroblasts by α-toxin. Furthermore, hemin-induced differentiation of human K562 erythroleukemia cells was impaired by α-toxin, whereas the treatment exhibited no apparent cytotoxicity. These results suggested that α-toxin mainly inhibited erythroid differentiation. Together, our results provide new insights into the biological activities of α-toxin, which might be important to understand the pathogenesis of *C. perfringens* infection.

## Introduction

*Clostridium perfringens* α-toxin, which is a major virulence factor during *C. perfringens* infection, is known to have two enzyme activities, phospholipase C (PLC) and sphingomyelinase (SMase), and these activities has been shown to be involved in various biological activities^1, 2^. Previously, it was reported that the toxin caused the contraction of rat ileum and aorta by activating phosphatidylinositol turnover and the production of thromboxane A_2_, respectively^3–5^. The toxin reduced skeletal muscle blood flow through thrombosis by promoting the aggregation of activated platelets and leukocytes, which caused the rapid destruction of skeletal muscle^6–8^. In addition, α-toxin has been reported to directly disrupt cell membrane and cause cytolysis^9–11^. These biological activities are proposed to play major roles in *C. perfringens*-induced myonecrosis. Recently, we reported that α-toxin inhibits neutrophil differentiation to impair the innate immune system by perturbing the integrity of lipid rafts in neutrophils^12, 13^. Thus, α-toxin affects a broad range of cell lines and induces various biological activities that are important to understand the pathogenesis of *C. perfringens* infection.

*C. perfringens* α-toxin is also known to induce the hemolysis of various erythrocytes. We reported that the toxin activated the sphingomyelin metabolic system leading to the hemolysis of sheep erythrocytes^14, 15^. Additionally, we reported previously that α-toxin activated endogenous PLC leading to the hemolysis of rabbit erythrocytes^16, 17^. In horse erythrocytes, α-toxin activates T-type Ca^2+^ channels leading to an increase in intracellular Ca^2+^, which plays an important role in hemolysis induced by the toxin^18^. Recently, *Bacillus anthracis* lethal toxin, which induces hemolysis *in vitro*, was reported to suppress erythropoiesis by killing erythroid progenitors and inhibit erythroid differentiation of cord blood-derived CD34^+^ hematopoietic stem cells^19^. The toxin-induced suppression of erythropoiesis seemed to be part of lethal toxin-mediated pathophysiology in anemia and hypoxia. This finding prompted us to hypothesize that *C. perfringens* α-toxin induces not only hemolysis but also dysfunction of erythropoiesis, which might be involved in the pathological process of *C. perfringens* infection.

In the present study, to clarify whether α-toxin affects erythroblasts, we treated isolated bone marrow cells (BMCs) from mice with purified α-toxin and found that TER119^+^ erythroblasts were greatly decreased by the treatment. Here, we demonstrate that *C. perfringens* α-toxin impairs erythroid differentiation, providing a new insight into the biological activities of α-toxin.

## Methods

### Mice

C57BL/6 J mice were purchased from Charles River Laboratories Japan, Inc., and were kept in a specific pathogen-free animal facility at Tokushima Bunri University. Animal experiments were approved by the Animal Care and Use Committee of Tokushima Bunri University, and procedures were performed in accordance with institutional guidelines, which conformed to the Fundamental Guidelines for Proper Conduct of Animal Experiment and Related Activities in Academic Research Institutions under the jurisdiction of the Ministry of Education, Culture, Sports, Science and Technology, 2006.

### Reagents

Fluorescein isothiocyanate (FITC)- or phycoerythrin (PE)-conjugated specific antibodies against mouse TER119 (clone TER-119) and mouse CD71 (clone C2F2), and purified rat anti-mouse CD16/CD32 (Fc Block) were purchased from BD Biosciences. Hemin was from Sigma-Aldrich. Recombinant human erythropoietin (EPO) was obtained from R&D systems. Cell counting kit-8 (CCK-8) was from Dojindo Molecular Technologies, Inc. Alexa Fluor 647-conjugated cholera toxin subunit B (CTB) was from Life Technologies. All other chemicals were of the highest grade available from commercial sources.

### Flow cytometry analysis

After blocking Fc-receptors with purified rat anti-mouse CD16/CD32, cells were labeled with the antibodies described above. Antibodies were diluted with phosphate-buffered saline (PBS) containing 2% fetal bovine serum (FBS; AusGeneX). To label apoptotic and necrotic cells, an Annexin-V-FLUOS staining kit (Roche Applied Science) was used. The labeled cells were analyzed using a Guava easyCyte (Millipore). FlowJo software (Tree Star) was used to analyze data.

### Preparation of bone marrow cells and cell culture

To obtain BMCs, femurs and tibias were crushed in PBS supplemented with 2% heat-inactivated FBS as described previously^12^. Briefly, red blood cells were hemolyzed with ACK lysing buffer (GIBCO) after the cells were filtered through a 40 µm mesh. The cells were stained with trypan blue to count the number of living cells. Isolated BMCs were cultured in RPMI 1640 medium supplemented with 10% FBS, 100 units/ml penicillin, and 100 µg/ml streptomycin in a humidified atmosphere of 95% air with 5% CO_2_ at 37 °C.

K562 human erythroleukemia cells were obtained from Riken Cell Bank (Tsukuba, Ibaraki, Japan). The cells were cultured in RPMI 1640 medium supplemented with 10% FBS, 100 units/ml penicillin, and 100 µg/ml streptomycin in a humidified atmosphere of 95% air with 5% CO_2_ at 37 °C. To induce the differentiation of K562 cells, the cells were cultured for 3–6 days in the presence of hemin, and hemoglobin synthesis in the cells was determined as reported previously^20^. Briefly, the cells were washed with cold PBS and suspended in lysis buffer (100 mM potassium phosphate pH 7.8, 0.2% Triton X-100). After the removal of cell debris, the supernatant was collected, and the hemoglobin concentration was determined by a 3,3′,5,5′-tetramethylbenzidine (TMB, Sigma-Aldrich) assay. The cell viability was determined using a cell counting kit-8 (CCK-8) (Dojindo Molecular Technologies, Inc.) in accordance with the manufacturer’s protocol.

### Purification of α-toxin

Purification of wild-type or H148G variant α-toxin was performed as described previously^21, 22^. Briefly, recombinant forms of pHY300PLK harboring the structural genes of wild-type or H148G variant α-toxin were introduced into *B. subtilis* ISW1214 by transformation. The transformants were cultured in Luria-Bertani broth containing 50 μg/ml ampicillin at 37 °C, and the culture medium was collected. Wild-type or H148G variant α-toxin secreted into the culture medium was purified chromatographically.

### Murine BFU-E/CFU-E assay

BMCs treated with 1, 10, or 100 ng/ml α-toxin for 24 hours in a humidified atmosphere of 95% air with 5% CO_2_ at 37 °C were washed and suspended in IMDM containing 10% FBS. The cells were then seeded in MethoCult M3334 (StemCell Technologies) and incubated for 2–10 days in a humidified atmosphere of 95% air with 5% CO_2_ at 37 °C in accordance with the manufacturer’s protocol. Subsequently, the number of colony-forming unit erythroid (CFU-E) and burst-forming unit erythroid (BFU-E) colonies were counted using a microscope.

### ELISA

Mice were injected intraperitoneally with 80 ng of purified α-toxin. Peripheral blood was obtained 24 hours after the injection via the vena cava using heparinized syringe. A mouse erythropoietin Quantikine ELISA kit (R&D Systems) was used to measure plasma EPO levels. The procedures were performed in accordance with the manufacturer’s instructions.

### Statistical analysis

All statistical analyses were performed with Easy R (Saitama Medical Center, Jichi Medical University)^23^. Differences between two groups were evaluated using two-tailed Student’s t-test. One-way analysis of variance (ANOVA) followed by the Tukey test was used to evaluate differences among three or more groups. Differences were considered to be significant for values of P < 0.05.

## Results

### α-Toxin decreases TER119^+^ erythroblasts in its enzyme activity-dependent manner

**Figure 1:**
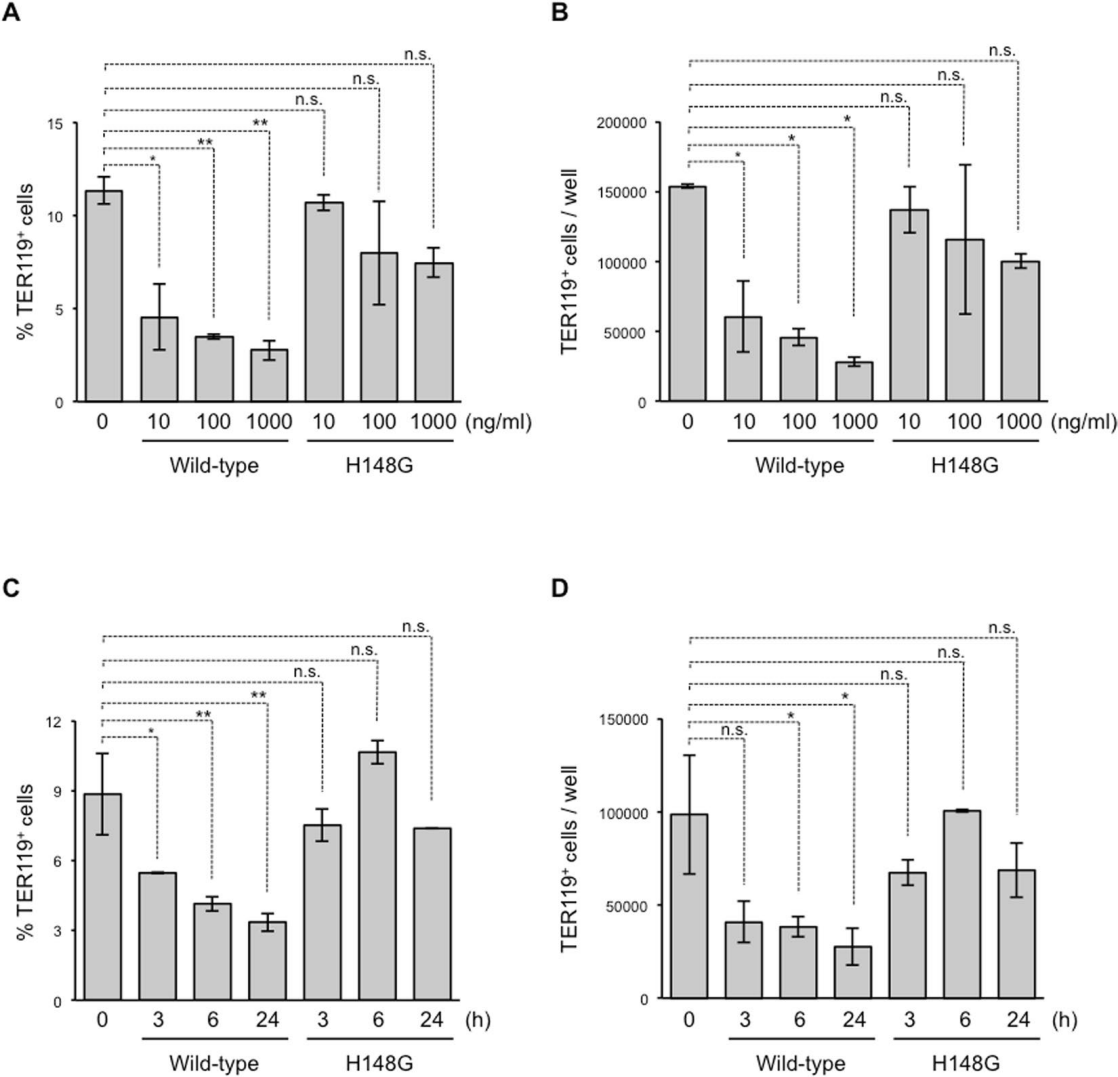
Treatment of bone marrow cells with α-toxin decreases TER119+ erythroblasts. Bone marrow cells were isolated from mice, and flow cytometry analysis was performed using a Guava easyCyte. (**A**,**B**) A total of 5 × 10^6^ bone marrow cells were cultured for 24 hours in the presence of the indicated concentration of α-toxin (Wild-type) or a variant α-toxin (H148G). The frequency (**A**) and absolute number of TER119^+^ cells per culture well (**B**) are shown. (**C**,**D**) Bone marrow cells were cultured for 3, 6, or 24 hours in the presence of 10 ng/ml α-toxin (Wild-type) or a variant α-toxin (H148G), and the frequency (**C**) and absolute number of TER119^+^ cells per culture well (**D**) are shown. One-way ANOVA was employed to assess statistical significance. Values are mean ± standard deviation. *P < 0.05; **P < 0.01; n.s., not significant.

Firstly, we isolated BMCs from C57BL/6 J mice and treated them with purified α-toxin for 24 h. As shown in Fig. 1A, a surface marker of murine erythroblasts, TER119^24, 25^, was expressed on around 10% of BMCs treated with control medium. Treatment of the cells with α-toxin greatly and dose-dependently decreased the proportion and number of TER119^+^ cells (Fig. 1A,B). Next, we treated the cells with α-toxin for 3, 6, or 24 h, and found that the decrease in TER119^+^ cells had already been induced after 3 h of treatment, and TER119^+^ cells decreased gradually up to 24 h, which meant that the reduction in TER119^+^ erythroblasts by α-toxin was time dependent (Fig. 1C,D). To test whether the enzyme activities of α-toxin were involved in the reduction of TER119^+^ cells, we used a variant α-toxin (H148G) defective in PLC and SMase activities^21^. The H148G variant α-toxin did not cause a decrease in TER119^+^ cells, suggesting that the enzyme activities play an important role in the reduction of erythroblasts by α-toxin (Fig. 1A–D).

### α-Toxin preferentially reduces mature erythroblasts

**Figure 2:**
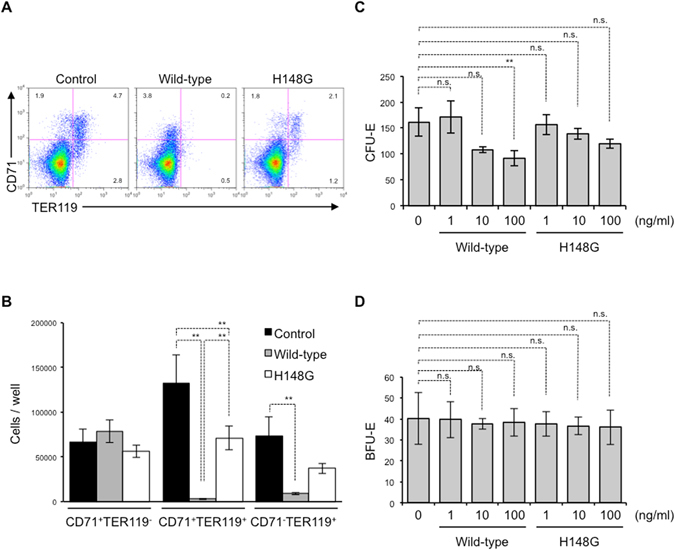
α-Toxin preferentially eradicates mature erythroblasts. (**A**,**B**) A total of 5 × 10^6^ bone marrow cells were cultured for 24 hours in the presence of 10 ng/ml α-toxin (Wild-type) or a variant α-toxin (H148G), and flow cytometry analysis was performed using a Guava easyCyte. (**A**) A representative flow cytometry profile is shown. (**B**) The absolute number of CD71^+^TER119^−^, CD71^+^TER119^+^, and CD71^−^TER119^+^ cells per culture well was determined. (**C**,**D**) Bone marrow cells were treated with the indicated concentration of α-toxin (Wild-type) or a variant α-toxin (H148G), and the cells were plated in Methocult 3334. CFU-E (**C**) and BFU-E (**D**) were determined. One-way ANOVA was employed to assess statistical significance. Values are mean ± standard deviation. **P < 0.01; n.s., not significant.

We performed detailed analysis of the erythroblast subpopulation by measuring cell-surface expression of CD71 and TER119 on BMCs treated with α-toxin. CD71^+^TER119^−^ cells containing mostly proerythroblasts and basophilic erythroblasts differentiated into CD71^+^TER119^+^ cells enriched for basophilic and polychromatophilic erythroblasts. Finally, CD71^+^TER119^+^ cells differentiated into CD71^−^TER119^+^ cells consisting mostly of normoblasts^26^. Wild-type and H148G variant α-toxin had no apparent effect on the proportion and number of CD71^+^TER119^−^ cells, whereas wild-type α-toxin greatly decreased those of CD71^+^TER119^+^ and CD71^−^TER119^+^ cells (Fig. 2A,B). The H148G variant α-toxin slightly reduced the number of CD71^+^TER119^+^ cells (Fig. 2B), so we tested whether the higher concentrations of the mutant toxin (1, 3 and 10 µg/ml) decreases erythroblasts. Figure S1 shows that the mutant toxin reduced the number of mature erythroblasts (CD71^+^TER119^+^ and CD71^−^TER119^+^ cells) but not immature cells (CD71^+^TER119^−^ cells) under these conditions. The activity of the mutant toxin decreased by approximately 100-fold of that of wild-type toxin (Fig. S1B,C). The enzyme activities, such as phospholipase C and sphingomyelinase, of H148G variant α-toxin are not completely eliminated by the mutation (Unpublished data), suggesting that the mutant’s residual activities could be involved in the decrease of TER119^+^ cells at the higher concentrations. Next, we measured the number of CFU-E colonies and more immature BFU-E colonies, and found that wild-type but not H148G variant α-toxin reduced the number of CFU-E colonies (Fig. 2C). On the other hand, α-toxin had no effect on the number of BFU-E colonies (Fig. 2D). These results demonstrated that α-toxin preferentially reduced mature erythroid cells compared with immature cells.

### α-Toxin impairs erythroid differentiation

**Figure 3:**
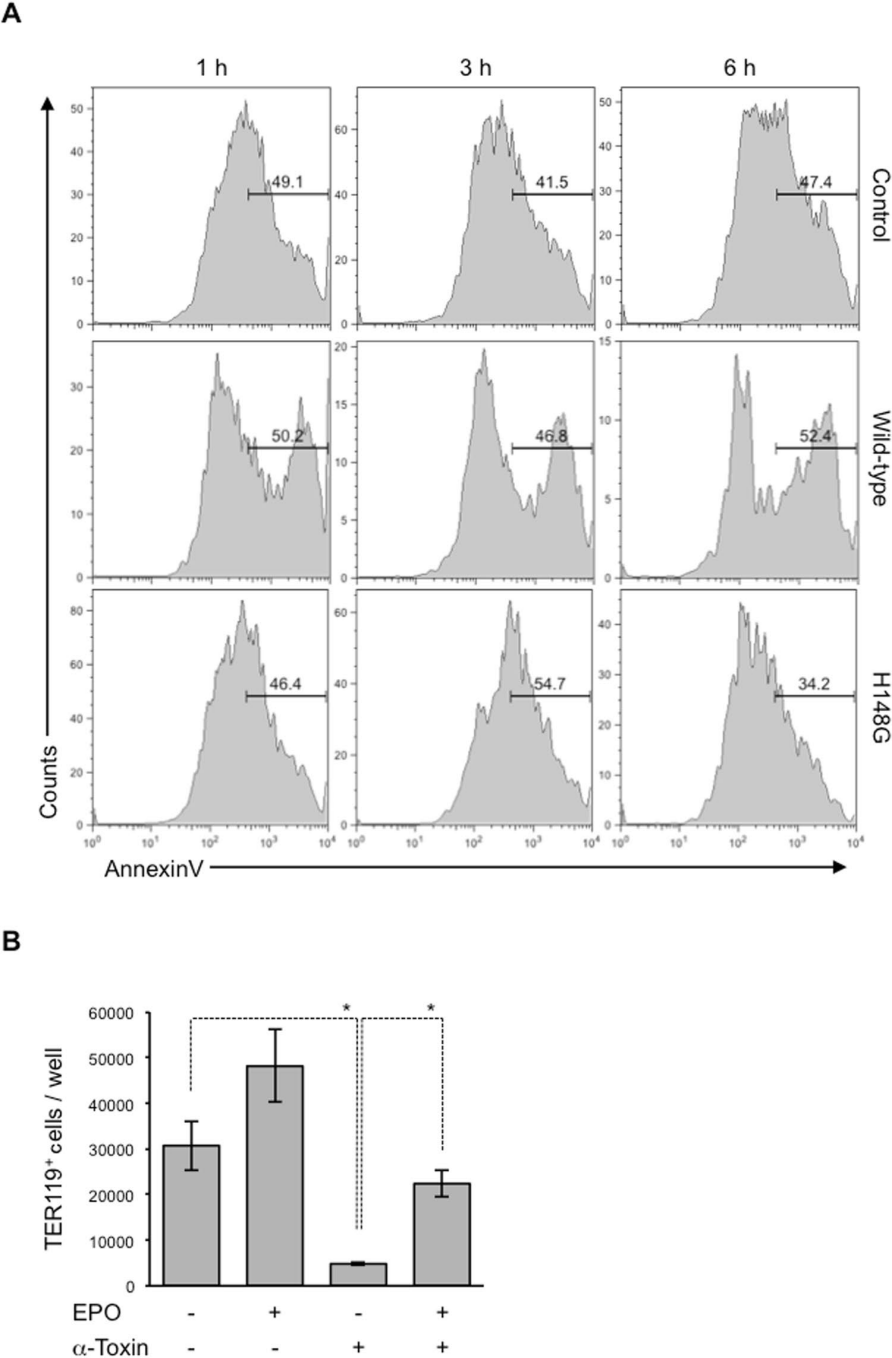
Erythropoietin attenuates the reduction in erythroblasts by α-toxin. (**A**) A total of 5 × 10^5^ bone marrow cells were cultured for 1, 3, or 6 hours in the presence or absence (Control) of 100 ng/ml α-toxin (Wild-type) or a variant α-toxin (H148G), and flow cytometry analysis was performed. The frequency of annexinV^+^ cells in the TER119^+^ cell population was determined. (**B**) A total of 5 × 10^6^ bone marrow cells were cultured for 24 hours in the presence of 10 ng/ml α-toxin (α-toxin) and 1 U/ml recombinant human EPO. The absolute number of TER119^+^ cells per culture well was determined by flow cytometry. One-way ANOVA was employed to assess statistical significance. Values are mean ± standard deviation. *P < 0.05.

At least two possible reasons for the decrease in TER119^+^ erythroblasts by α-toxin were suggested. One was that the toxin led to cell death in TER119^+^ cells, and the other was that it blocks the differentiation of immature erythroblasts, such as proerythroblasts and basophilic erythroblasts. To identify the mechanisms by which α-toxin reduces TER119^+^ erythroblasts, we stained BMCs treated with α-toxin with FITC-conjugated annexinV, and measured the proportion of annexinV^+^ apoptotic and necrotic cells in TER119^+^ cells. α-Toxin slightly increased annexinV^+^ cells in TER119^+^ cells, showing that α-toxin had limited cytotoxicity with TER119^+^ erythroblasts (Fig. 3A). Next, simultaneous treatment of BMCs with α-toxin and recombinant EPO, a well-known cytokine that mediates erythroid differentiation^27^, was performed. The treatment with EPO greatly attenuated the reduction in TER119^+^ cells by α-toxin (Fig. 3B). These results suggested that blockage of erythroid differentiation was also involved in the reduction of TER119^+^ erythroblasts by α-toxin.

**Figure 4:**
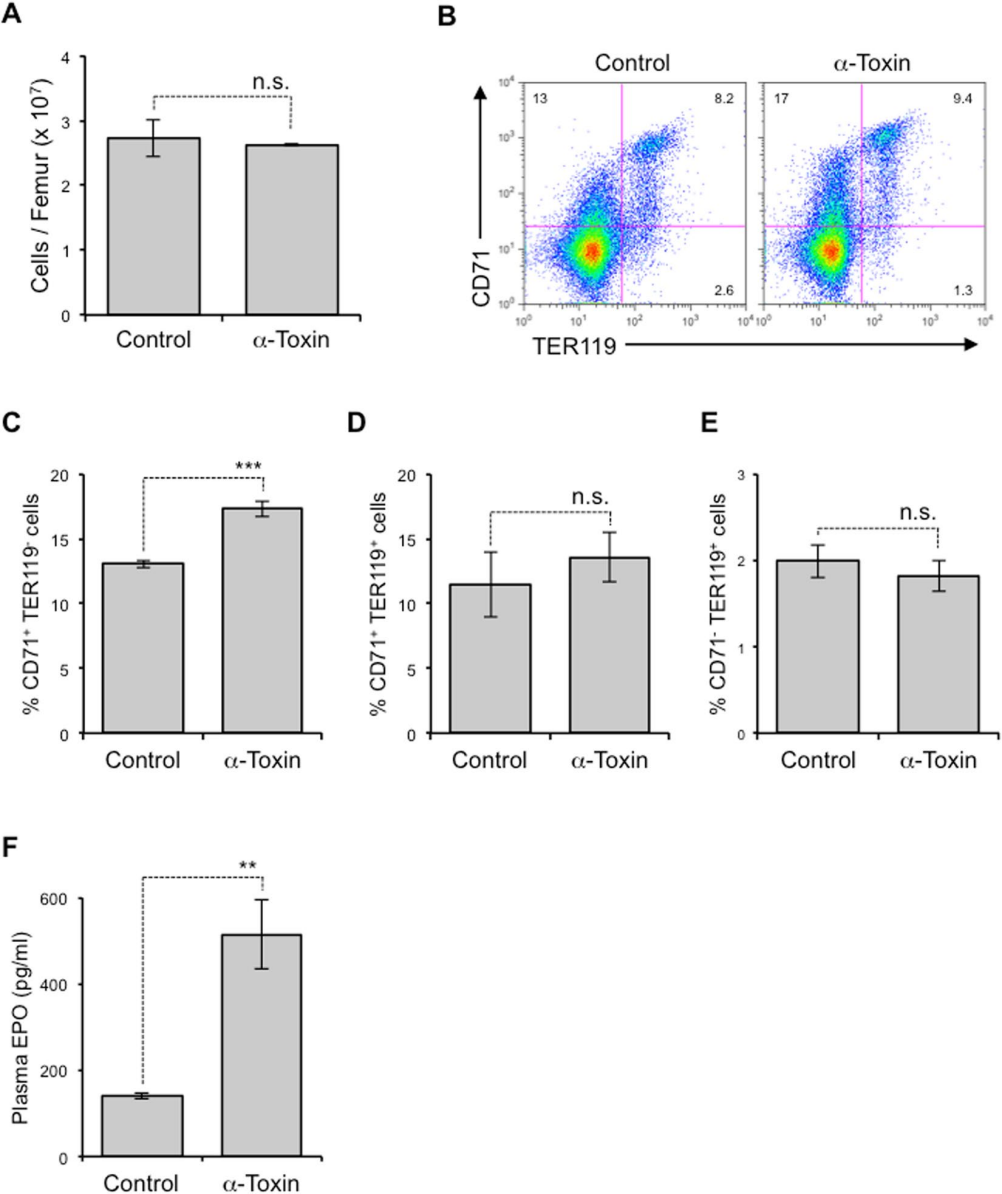
The *in vivo* effect of α-toxin on erythropoiesis. Mice were injected intraperitoneally with 80 ng of purified α-toxin (α-Toxin) or PBS as a control (Control). BMCs and peripheral blood were isolated 24 hours after the injection. (**A**) Bone marrow cellularity was determined. (**B**–**E**) Flow cytometry analysis of BMCs was performed using specific antibodies against CD71 and TER119. A representative flow cytometry profile (**B**), the frequency of CD71^+^TER119^−^ cells (**C**), CD71^+^TER119^+^ cells (**D**), and CD71^−^TER119^+^ cells (**E**) are shown. (**F**) Plasma EPO level was measured by ELISA. Two-tailed Student’s t-test was employed to assess statistical significance. Values are mean ± standard error. **P < 0.01; ***P < 0.001; n.s., not significant.

To examine whether α-toxin affects erythropoiesis *in vivo*, we intraperitoneally injected a sub-lethal dose of α-toxin (80 ng per mouse) to mice and quantified erythroblasts. The injection had no profound impact on the bone marrow cellularity (Fig. 4A), while it increased the proportion of immature erythroblasts (CD71^+^TER119^−^ cells) but not mature cells (CD71^+^TER119^+^ and CD71^−^TER119^+^ cells) (Fig. 4B–E). Surprisingly, the injection increased plasma EPO, a well-known cytokine that mediates erythroid differentiation^28^ (Fig. 4F). As shown in Fig. 3B, the treatment with EPO greatly attenuated the reduction in TER119^+^ cells by α-toxin. Taken together, our results suggest that the increase of plasma EPO might mask the inhibitory effects on erythroid differentiation by α-toxin *in vivo*.

**Figure 5:**
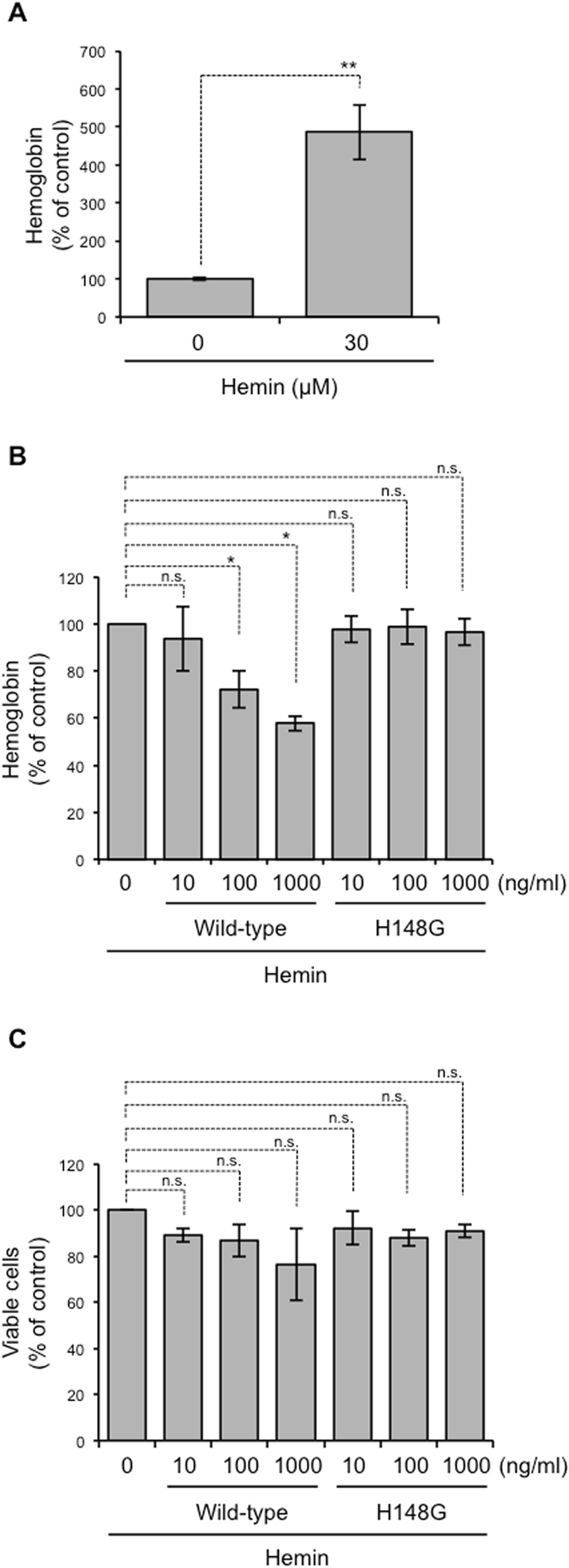
α-Toxin impairs differentiation of K562 cells. K562 cells were cultured in the presence of 30 μM hemin. Simultaneously, the cells were treated with 10 ng/ml α-toxin (Wild-type) or a variant α-toxin (H148G). (**A**,**B**) Hemoglobin synthesis in K562 cells was determined using a TMB assay. The relative amounts of hemoglobin are expressed as a percentage relative to the control cells. (**C**) The cell viability was evaluated using a CCK-8 assay. The relative cell viabilities are expressed as a percentage relative to α-toxin-untreated control cells. One-way ANOVA and two-tailed Student’s t-test were employed to assess statistical significance. Values are mean ± standard deviation. *P < 0.05; **P < 0.01; n.s., not significant.

The sensitivity of erythrocytes to hemolysis by α-toxin varies markedly in different species. Human and rabbit erythrocytes can be easily lysed by the toxin, whereas hemolysis of equine erythrocytes requires a higher concentration of α-toxin^29^. The difference has been proposed to depend on diversity of phospholipid composition of the cell membrane^10^. α-Toxin can hydrolyze both phosphatidylcholine (PC) and sphingomyelin (SM) from erythrocyte membranes, and the proportion of these phospholipids seems to affect a tolerance of erythrocytes against the α-toxin-induced hemolysis. Mouse erythrocytes possess similar proportions of PC and SM in the outer membrane leaflet whereas horse erythrocytes have a higher proportion of PC^30^. To elucidate whether α-toxin impairs the differentiation of human erythroid progenitors, we treated K562 human erythroleukemia cells with α-toxin while inducing the differentiation of cells using hemin, and monitored the erythroid differentiation by measuring hemoglobin production. As described previously^20^, hemin increased the production of hemoglobin, demonstrating that the compound induces erythroid differentiation of K562 cells (Fig. 5A). Wild-type but not H148G variant α-toxin decreased the production of hemoglobin in cells treated with hemin (Fig. 5B). In addition, α-toxin had no apparent cytotoxicity with hemin-treated K562 cells (Fig. 5C). Taken together, our results suggest that α-toxin inhibits the differentiation of human erythroid progenitors, which might be important to understand the pathogenesis of *C. perfringens* infection in humans.

### Molecular mechanism of α-toxin-mediated blockage of erythroid differentiation

**Figure 6:**
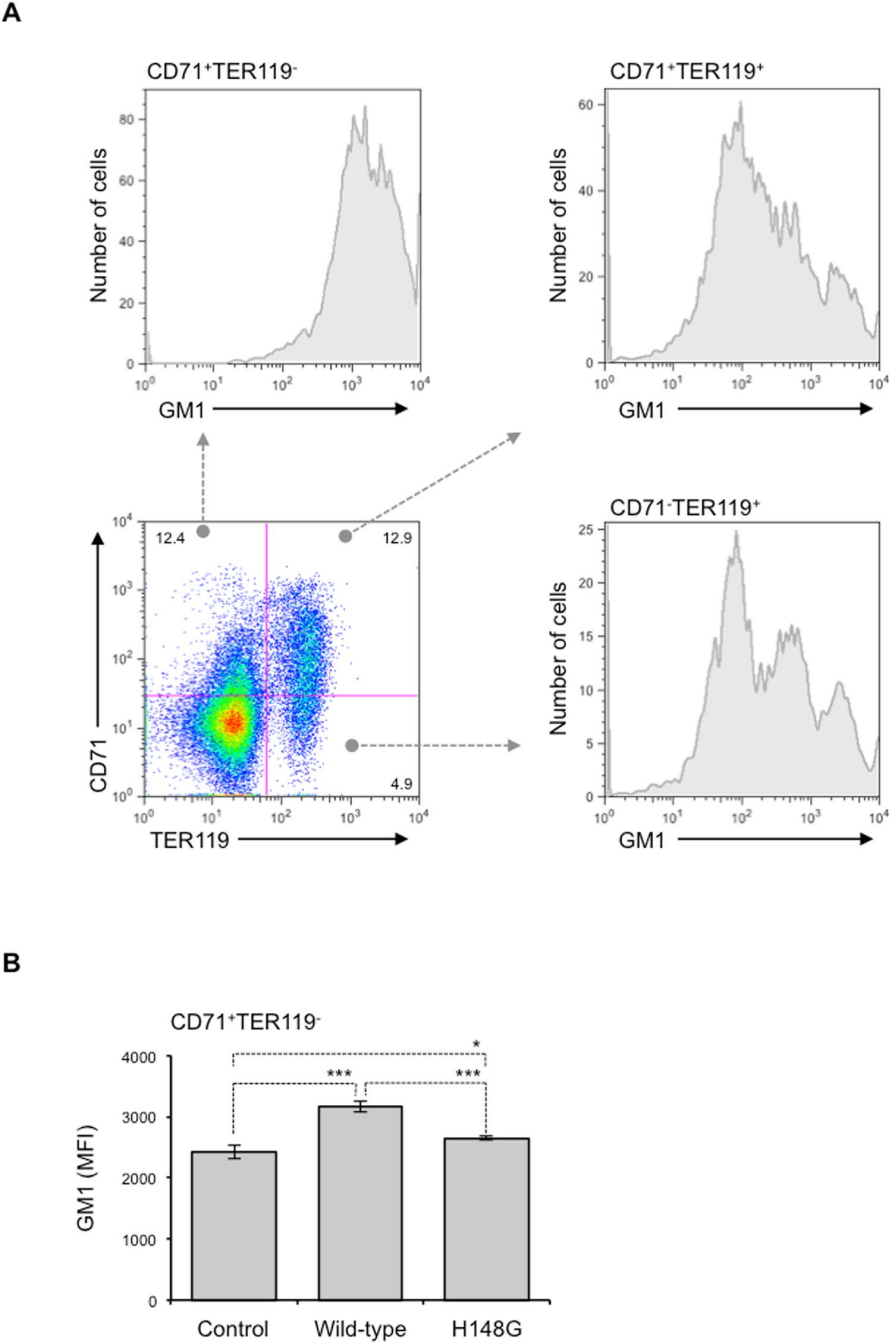
α**-**Toxin increases expression of GM1 in erythroblasts. (**A**) BMCs were labeled with Alexa Fluor 647-CTB and specific antibodies against CD71 and TER119. The cells were analyzed using a Guava easyCyte, and fluorescence of Alexa Fluor 647-CTB in three different populations (CD71^+^TER119^−^, CD71^+^TER119^+^, and CD71^−^TER119^+^ cells) was determined. (**B**) A total of 5 × 10^6^ bone marrow cells were treated with 100 ng/ml α-toxin (Wild-type) or a variant α-toxin, H148G (H148G), for 24 hours, and the cells were labeled as described above. Fluorescence of Alexa Fluor 647-CTB in the CD71^+^TER119^−^ cell population was determined, and the mean fluorescence intensity was compared with that of α-toxin-untreated cells (Control). One-way ANOVA was employed to assess statistical significance. Values are mean ± standard deviation. *P < 0.05; ***P < 0.001.

Recently, we reported that the integrity of lipid rafts should be properly maintained during neutrophil differentiation, and that the perturbation of lipid raft integrity by α-toxin or a lipid raft-disrupting agent, methyl-β-cyclodextrin, resulted in impairment of neutrophil differentiation^13^. Moreover, the clustering of lipid rafts at the furrow between incipient reticulocytes and pyrenocytes was reported to be necessary during erythroblast enucleation^31^, suggesting that lipid rafts play an important role in the regulation of erythropoiesis. To test whether integrity of lipid rafts should be properly maintained during erythroid differentiation, we quantified the cell surface expression of a lipid raft marker, GM1 ganglioside (GM1)^32^, in several different erythroid lineages from naive mice by using CTB that specifically bind to GM1^33^. The cell surface expression of GM1 decreased in association with erythroid differentiation (Fig. 6A). Figure 6B shows that α-toxin treatment increased the expression of GM1 in CD71^+^TER119^−^ cells, suggesting that the toxin affects lipid raft integrity in erythroid progenitors.

Next, we examined whether phosphorylation of signaling molecules already known to be activated by α-toxin plays a part in the differentiation blockage of K562 cells. Phosphoinositide 3-kinase (PI3K), phospholipase C γ-1 (PLCγ1), nuclear factor κB (NF-κB), extracellular signal-regulated kinases (ERK) 1/2 and p38 mitogen-activated protein kinase (MAPK) have been reported to be phosphorylated and activated by α-toxin^34–37^. As shown in Fig. S2, α-toxin did not induce the phosphorylation of these molecules in K562 cells, suggesting that these signal transduction molecules are not involved in the α-toxin-induced differentiation blockage.

## Discussion

*C. perfringens* α-toxin, which is a major virulence factor during *C. perfringens* infection, has been shown to possess various biological activities in inducing hemolysis, cytolysis, and thrombosis^1, 2^. These activities are important to understand the pathogenesis of *C. perfringens* infection. Recently, we reported that the toxin impairs granulopoiesis to disturb the innate immune system^12^, which could contribute to the characteristics of *C. perfringens* infection such as an absence of polymorphonuclear leukocytes at the site of infection^38, 39^. Thus, α-toxin inhibits the differentiation of neutrophils, but it has not been elucidated whether the toxin affects the differentiation of other myeloid cell types. In the present study, we treated isolated BMCs with α-toxin and found that mature erythroblasts were greatly and preferentially decreased by this treatment. The subsequent experiments revealed that blockage of erythroid differentiation was involved in the reduction of TER119^+^ erythroblasts by α-toxin. Clinically, massive intravascular hemolysis, sometimes leading to anemia, has been reported to occur in *C. perfringens*-infected patients^40^. Hashiba *et al*. reported that severe anemia was seen in a patient infected with *C. perfringens* ^41^. The detailed mechanisms by which *C. perfringens* infection causes severe anemia has not been elucidated, but taking account of not only hemolysis but blockage of erythropoiesis by α-toxin might be necessary for treating *C. perfringens* infection. Further experiments would be needed to investigate the clinical aspect how the blockage of erythropoiesis is involved in the pathogenesis of *C. perfringens* infection.

Experiments using the H148G variant α-toxin, which lacks enzyme activities such as PLC and SMase^21^, revealed that the activities play an important role in the reduction of erythroblasts by α-toxin. Diaz *et al*. reported that *Staphylococcus aureus* SMase disrupts cholesterol-rich plasma membrane microdomains, so-called lipid rafts, in human lymphocytes^42^. Recently, we reported that *C. perfringens* α-toxin disturbs lipid raft integrity in neutrophils in an enzyme activity-dependent manner^13^. Lipid rafts act as platforms for signaling molecules that regulate cell differentiation^43^. For instance, glial cell line-derived neurotrophic factor (GDNF) family ligands stimulate GDNF receptor-α and recruit transmembrane tyrosine kinase to lipid rafts, leading to the activation of intracellular signaling events involved in the development of the nervous system^44^. We also reported that the integrity of lipid rafts should be properly maintained during neutrophil differentiation, and that the perturbation of lipid raft integrity by a lipid raft-disrupting agent, methyl-β-cyclodextrin, resulted in the impairment of neutrophil differentiation^13^. Moreover, the clustering of lipid rafts at the furrow between incipient reticulocytes and pyrenocytes was reported to be necessary during erythroblast enucleation, suggesting that lipid rafts play an important role in the regulation of erythropoiesis^31^. Thus, lipid rafts play important roles in the differentiation of many types of cell. In the present study, we revealed that the cell surface expression of a lipid raft marker, GM1, decreased in association with erythroid differentiation, and that α-toxin affected lipid raft integrity in erythroid progenitors. The detailed mechanism by which the disturbance in the lipid raft integrity impairs erythroid differentiation remains unclear, but our results give a new insight on the regulation of erythriod differentiation. Because SMase is known to hydrolyze cell membrane sphingomyelin to ceramide, which is a lipid raft component^45, 46^, α-toxin might cause the overproduction of ceramide in erythroblasts leading to the disturbance of lipid raft integrity, and mediate the blockage of erythroid differentiation. In addition, ceramide is known to work as a lipid messenger mediating various cellular responses including cell differentiation^47^. The other possible explanation for the α-toxin-induced blockage of erythroid differentiation is that the accelerated production of ceramide by the toxin modifies ceramide signaling.

Crystal structure analysis of α-toxin revealed that it consists of two domains, the *N*-domain and *C*-domain^48^. The catalytic domain of α-toxin is located in the *N*-domain, whereas the *C*-domain is supposed to play an important role in binding to the cell membrane^2, 49, 50^. Additionally, a loop region in the *N*-domain contributes to binding of the toxin to GM1a ganglioside^51^. Recently, we reported that cell surface expression of GM1 ganglioside was barely detected in TER119^+^ erythroblasts^13^. These observations suggest that the *C*-domain but not the loop region is indispensable for the interaction between α-toxin and erythroblasts.

Here, we failed to detect the inhibitory effects by α-toxin on erythroid differentiation *in vivo*. Surprisingly, injection of α-toxin greatly increased plasma EPO, suggesting that the increase of plasma EPO might mask the α-toxin-induced inhibitory effect on erythroid differentiation under our experimental conditions. The increase of plasma EPO might be explained by a renal compensatory mechanism that responses to mild or almost undetectable hemolysis^52^. It would be important to evaluate the effects under *C. perfringens* infection because the other toxins might collaborate with α-toxin to impair erythropoiesis *in vivo*. For examples, we reported that *C. perfringens* ε-toxin and δ-toxin induced cell death of Madin-Darby Canine Kidney (MDCK) cells^53, 54^, suggesting that these toxins might cause renal damage. In such conditions, α-toxin might exert a cumulative effect on virulence against erythropoiesis. Further investigation should be necessary to elucidate whether α-toxin impairs erythropoiesis in clinical settings.

In conclusion, α-toxin inhibited erythroid differentiation, resulting in a reduction in erythroblasts. Our data provide new insights into the biological activities of α-toxin, which might be pivotal for comprehending the pathogenesis of *C. perfringens* infection, and offer a deeper understanding of α-toxin-mediated host-pathogen interactions.

## Electronic supplementary material

Supplementary Figure S1, Figure S2, and Methods

## References

[1] Bryant AE (2003). Biology and pathogenesis of thrombosis and procoagulant activity in invasive infections caused by group A streptococci and *Clostridium perfringens*. Clin Microbiol Rev..

[2] Sakurai J, Nagahama M, Oda M (2004). *Clostridium perfringens* alpha-toxin: characterization and mode of action. J Biochem..

[3] Fujii Y, Nomura S, Oshita Y, Sakurai J (1986). Excitatory effect of *Clostridium perfringens* alpha toxin on the rat isolated aorta. Br J Pharmacol..

[4] Fujii Y, Sakurai J (1989). Contraction of the rat isolated aorta caused by *Clostridium perfringens* alpha toxin (phospholipase C): evidence for the involvement of arachidonic acid metabolism. Br J Pharmacol..

[5] Sakurai J, Fujii Y, Shirotani M (1990). Contraction induced by *Clostridium perfringens* alpha toxin in the isolated rat ileum. Toxicon.

[6] Bryant AE (2000). Clostridial gas gangrene. II. Phospholipase C-induced activation of platelet gpIIbIIIa mediates vascular occlusion and myonecrosis in *Clostridium perfringens* gas gangrene. J Infect Dis..

[7] Hickey MJ (2008). Molecular and cellular basis of microvascular perfusion deficits induced by *Clostridium perfringens* and *Clostridium septicum*. PLoS Pathog..

[8] Bunting M (1997). Alpha toxin from *Clostridium perfringens* induces proinflammatory changes in endothelial cells. J Clin Invest..

[9] Nagahama M, Michiue K, Sakurai J (1996). Membrane-damaging action of *Clostridium perfringens* alpha-toxin on phospholipid liposomes. Biochim Biophys Acta.

[10] Flores-Diaz M, Thelestam M, Clark GC, Titball RW, Alape-Giron A (2004). Effects of *Clostridium perfringens* phospholipase C in mammalian cells. Anaerobe.

[11] Flores-Diaz M (2005). A cellular deficiency of gangliosides causes hypersensitivity to *Clostridium perfringens* phospholipase C. J Biol Chem..

[12] Takehara M (2016). *Clostridium perfringens* alpha-Toxin Impairs Innate Immunity via Inhibition of Neutrophil Differentiation. Sci Rep..

[13] Takehara M (2016). *Clostridium perfringens* alpha-Toxin Impairs Lipid Raft Integrity in Neutrophils. Biol Pharm Bull.

[14] Ochi S, Oda M, Matsuda H, Ikari S, Sakurai J (2004). *Clostridium perfringens* alpha-toxin activates the sphingomyelin metabolism system in sheep erythrocytes. J Biol Chem..

[15] Oda M (2008). The relationship between the metabolism of sphingomyelin species and the hemolysis of sheep erythrocytes induced by *Clostridium perfringens* alpha-toxin. J Lipid Res.

[16] Sakurai J, Ochi S, Tanaka H (1994). Regulation of *Clostridium perfringens* alpha-toxin-activated phospholipase C in rabbit erythrocyte membranes. Infect Immun..

[17] Sakurai J, Ochi S, Tanaka H (1993). Evidence for coupling of *Clostridium perfringens* alpha-toxin-induced hemolysis to stimulated phosphatidic acid formation in rabbit erythrocytes. Infect Immun..

[18] Ochi S, Oda M, Nagahama M, Sakurai J (2003). *Clostridium perfringens* alpha-toxin-induced hemolysis of horse erythrocytes is dependent on Ca2+ uptake. Biochim Biophys Acta.

[19] Chang HH (2013). Erythropoiesis suppression is associated with anthrax lethal toxin-mediated pathogenic progression. PLoS One.

[20] Zhang D, Cho E, Wong J (2007). A critical role for the co-repressor N-CoR in erythroid differentiation and heme synthesis. Cell Res..

[21] Nagahama M, Okagawa Y, Nakayama T, Nishioka E, Sakurai J (1995). Site-directed mutagenesis of histidine residues in *Clostridium perfringens* alpha-toxin. J Bacteriol..

[22] Nagahama M (2013). A recombinant carboxy-terminal domain of alpha-toxin protects mice against *Clostridium perfringens*. Microbiol Immunol..

[23] Kanda Y (2013). Investigation of the freely available easy-to-use software ‘EZR’ for medical statistics. Bone Marrow Transplant..

[24] Ikuta K (1990). A developmental switch in thymic lymphocyte maturation potential occurs at the level of hematopoietic stem cells. Cell.

[25] Kina T (2000). The monoclonal antibody TER-119 recognizes a molecule associated with glycophorin A and specifically marks the late stages of murine erythroid lineage. Br J Haematol..

[26] Marinkovic D (2007). Foxo3 is required for the regulation of oxidative stress in erythropoiesis. J Clin Invest..

[27] Lacombe C, Mayeux P (1998). Biology of erythropoietin. Haematologica.

[28] Chateauvieux S, Grigorakaki C, Morceau F, Dicato M, Diederich M (2011). Erythropoietin, erythropoiesis and beyond. Biochem Pharmacol..

[29] Ikezawa H, Murata R (1964). Comparative Kinetics of Hemolysis of Mammalian Erythrocytes by *Clostridium Perfringens* Alpha-Toxin (Phospholipase C). J Biochem..

[30] Jepson M, Titball R (2000). Structure and function of clostridial phospholipases C. Microbes Infect..

[31] Konstantinidis DG (2012). Signaling and cytoskeletal requirements in erythroblast enucleation. Blood.

[32] Brown DA, London E (2000). Structure and function of sphingolipid- and cholesterol-rich membrane rafts. J Biol Chem..

[33] Blank N (2007). Cholera toxin binds to lipid rafts but has a limited specificity for ganglioside GM1. Immunol Cell Biol..

[34] Oda M (2006). Signal transduction mechanism involved in *Clostridium perfringens* alpha-toxin-induced superoxide anion generation in rabbit neutrophils. Infect Immun..

[35] Takagishi, T. *et al*. *Clostridium perfringens* Alpha-Toxin Induces Gm1a Clustering and Trka Phosphorylation in the Host Cell Membrane. *PLoS One* **10** (2015). 10.1371/journal.pone.0120497 PMC4409118 25910247

[36] Monturiol-Gross L, Flores-Diaz M, Pineda-Padilla MJ, Castro-Castro AC, Alape-Giron A (2014). *Clostridium perfringens* phospholipase C induced ROS production and cytotoxicity require PKC, MEK1 and NFkappaB activation. PLoS One.

[37] Oda M (2012). *Clostridium perfringens* alpha-toxin induces the release of IL-8 through a dual pathway via TrkA in A549 cells. Biochim Biophys Acta.

[38] Stevens DL, Tweten RK, Awad MM, Rood JI, Bryant AE (1997). Clostridial gas gangrene: evidence that alpha and theta toxins differentially modulate the immune response and induce acute tissue necrosis. J Infect Dis..

[39] Bahl, H. & Dürre, P. Clostridia: biotechnology and medical applications. (Wiley-VCH, 2001).

[40] Kurasawa M, Nishikido T, Koike J, Tominaga S, Tamemoto H (2014). Gas-forming liver abscess associated with rapid hemolysis in a diabetic patient. World J Diabetes.

[41] Hashiba M (2016). *Clostridium Perfringens* Infection in a Febrile Patient with Severe Hemolytic Anemia. Am J Case Rep..

[42] Diaz O (2005). Disruption of lipid rafts stimulates phospholipase d activity in human lymphocytes: implication in the regulation of immune function. J Immunol..

[43] Simons K, Toomre D (2000). Lipid rafts and signal transduction. Nat Rev Mol Cell Biol..

[44] Tansey MG, Baloh RH, Milbrandt J, Johnson EM (2000). GFRalpha-mediated localization of RET to lipid rafts is required for effective downstream signaling, differentiation, and neuronal survival. Neuron.

[45] Milhas D, Clarke CJ, Hannun YA (2010). Sphingomyelin metabolism at the plasma membrane: implications for bioactive sphingolipids. FEBS Lett..

[46] Bollinger CR, Teichgraber V, Gulbins E (2005). Ceramide-enriched membrane domains. Biochim Biophys Acta.

[47] Kolesnick RN, Kronke M (1998). Regulation of ceramide production and apoptosis. Annu Rev Physiol..

[48] Naylor CE (1998). Structure of the key toxin in gas gangrene. Nat Struct Biol..

[49] Oda M, Terao Y, Sakurai J, Nagahama M (2015). Membrane-Binding Mechanism of *Clostridium perfringens* Alpha-Toxin. Toxins (Basel).

[50] Nagahama M, Mukai M, Morimitsu S, Ochi S, Sakurai J (2002). Role of the C-domain in the biological activities of *Clostridium perfringens* alpha-toxin. Microbiol Immunol..

[51] Oda M (2012). Clostridium perfringens alpha-toxin recognizes the GM1a-TrkA complex. J Biol Chem..

[52] Jelkmann W (1992). Erythropoietin: structure, control of production, and function. Physiol Rev..

[53] Takagishi T, Oda M, Takehara M, Kobayashi K, Nagahama M (2016). Oligomer formation of *Clostridium perfringens* epsilon-toxin is induced by activation of neutral sphingomyelinase. Biochim Biophys Acta.

[54] Seike S, Miyamoto K, Kobayashi K, Takehara M, Nagahama M (2016). *Clostridium perfringens* Delta-Toxin Induces Rapid Cell Necrosis. PLoS One.

