# Supplementary material for: Clostridium perfringens α-toxin impairs erythropoiesis by inhibition of erythroid differentiation
Source: Sci Rep. 2017 Jul 12;7:5217. doi: 10.1038/s41598-017-05567-8 (PMC5507896; doi:10.1038/s41598-017-05567-8)
Supplement: Supplementary file 1 — Supplementary Figure S1, Figure S2, and Methods [file 41598_2017_5567_MOESM1_ESM.pdf]

Clostridium perfringens α-toxin impairs erythropoiesis by inhibition of erythroid differentiation

Teruhisa Takagishi[a], Masaya Takehara[a,*], Soshi Seike, Kazuaki Miyamoto, Keiko Kobayashi, Masahiro Nagahama*

Department of Microbiology, Faculty of Pharmaceutical Sciences, Tokushima Bunri University, Yamashiro-cho, Tokushima 770-8514, Japan

*To whom correspondence should be addressed: Dr. Masaya Takehara and Dr. Masahiro Nagahama, Department of Microbiology, Faculty of Pharmaceutical Sciences, Tokushima Bunri University, Yamashiro-cho, Tokushima 770-8514, Japan; E-mail: mtakehara@ph.bunri-u.ac.jp and nagahama@ph.bunri-u.ac.jp; Tel.: +81-88-602-8485, Fax: +81-88-655-3051.

[a] These authors contributed equally to this work.

Table of Contents

Supplementary Figures S1 and S2

Supplementary Methods

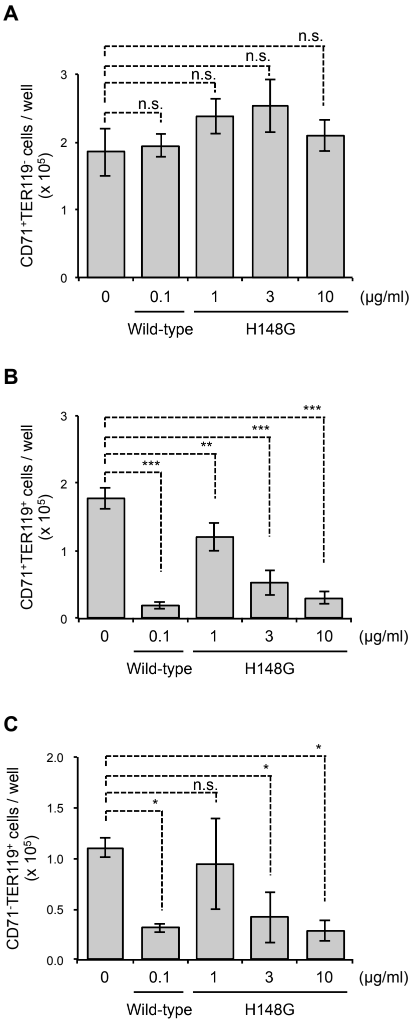

Figure S1. Treatment of BMCs with high-dose H148G variant α-toxin decreases mature erythroblasts. A total of 5×10^6 bone marrow cells were cultured for 24 hours in the presence of the indicated concentration of α-toxin (Wild-type) or a variant α-toxin (H148G), and flow cytometry analysis was performed using a Guava easyCyte. The absolute number of $CD71^+TER119^-$ (A), $CD71^+TER119^+$ (B), and $CD71^-TER119^+$ cells (C) per culture well was determined.

One-way ANOVA was employed to assess statistical significance. Values are mean ± standard deviation. $^{*}P < 0.05$; $^{**}P < 0.01$; $^{***}P < 0.001$; n.s., not significant.

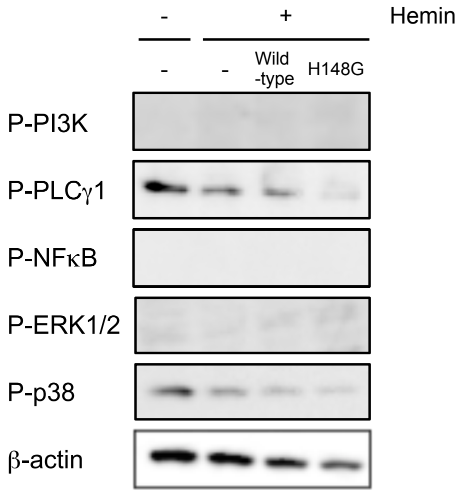

Figure S2. Phosphorylation of signaling molecules already known to be activated by α-toxin. K562 cells were cultured in the presence of 30 μM hemin. Simultaneously, the cells were treated with 100 ng/ml α-toxin (Wild-type) or a variant α-toxin, H148G (H148G) for 3 days. Whole cell extracts from the cells were analyzed by immunoblotting with phospho-specific antibodies against PI3K (P-PI3K), PLCγ1 (P-PLCγ1), NF-κB (P-NFκB), ERK 1/2 (P-ERK1/2) or p38 MAPK (P-p38), or a specific antibody against β-actin (β-actin).

Supplementary Methods

Reagents. Phospho-specific antibodies against PI3K, PLCγ1, NF-κB, ERK 1/2, and p38 MAPK were from Cell Signaling Technology. An antibody against β-actin was purchased from Santa Cruz Biotechnology.

Immunoblotting analysis. Immunoblotting analysis was performed as previously described with some modifications[55]. Briefly, cells were lysed in RIPA buffer (Nacalai Tesque). The protein concentrations of the samples were determined using a BCA protein assay kit (Nacalai Tesque). Samples were applied to 10% polyacrylamide gels containing SDS, subjected to electrophoresis, and transferred to a polyvinylidene difluoride membrane (PVDF, Immobilon P; Millipore). The membrane was blocked with Blocking One-P (Nacalai Tesque), and the proteins were then immunoblotted with each antibody.

Reference

55 Seike, S., Takehara, M., Kobayashi, K. & Nagahama, M. Role of pannexin 1 in *Clostridium perfringens* beta-toxin-caused cell death. *Biochim Biophys Acta* **1858**, 3150-3156 (2016)
